# The Congenital Heart Disease in Children: Kidney-Associated Conditions with Epidemiologic Endpoints (CHICKADEE) study: Study design and protocol

**DOI:** 10.1371/journal.pone.0356639

**Published:** 2026-08-20

**Authors:** Jason H. Greenberg, Derek K. Ng, Eyal Sagiv, Mark Mitsnefes, Addison Gearhart, Lucy Mulqueen, Olyvia J. Hanken-Arlen, Henon Gebre, Ilham Boutahri, Allen D. Everett, Joseph T. Flynn, Chirag R. Parikh

**Affiliations:** 1 Division of Nephrology, Department of Pediatrics, Yale University School of Medicine, New Haven, Connecticut, United States of America; 2 Department of Medicine, Clinical and Translational Research Accelerator, Yale University School of Medicine, New Haven, Connecticut, United States of America; 3 Department of Epidemiology, Johns Hopkins Bloomberg School of Public Health, Baltimore, Maryland, United States of America; 4 Division of Nephrology, Johns Hopkins University School of Medicine, Baltimore, Maryland, United States of America; 5 Division of Cardiology, Department of Pediatrics, University of Washington and Seattle Children’s Hospital, Seattle, Washington, United States of America; 6 Division of Nephrology, Cincinnati Children’s Hospital Medical Center, Cincinnati, Ohio, United States of America; 7 Division of Pediatric Cardiology, Department of Pediatrics, Johns Hopkins University, Baltimore, Maryland, United States of America; 8 Division of Nephrology, Department of Pediatrics, University of Washington and Seattle Children’s Hospital, Seattle, Washington, United States of America; PLOS: Public Library of Science, UNITED KINGDOM OF GREAT BRITAIN AND NORTHERN IRELAND

## Abstract

**Background:**

Congenital heart disease (CHD) is the most common structural birth defect affecting 1% of live births. With improvements in medical and surgical management, there are now more than 2 million children and adults in the United States with CHD, a number that continues to grow. Children who undergo cardiac surgery for CHD face elevated risks of hypertension, chronic kidney disease (CKD), kidney failure, and premature mortality—complications that are more prevalent in those with single ventricle physiology and increase as they get older. Although emerging evidence suggests that the excess burden of CKD observed in adults with CHD originates in childhood, these complications remain under-recognized because they are understudied in pediatric populations and represent a critical missed opportunity for evaluation and early intervention.

**Objectives:**

This multi-center prospective cohort study aims to characterize the epidemiology, risk factors, and mechanisms of CKD and hypertension in children several years after CHD surgery, with a particular focus on complex CHD conditions.

**Methods:**

We aim to enroll 300 children, 4 to 16 years old, across three clinical sites, 4 to 12 years after their initial CHD surgery. The cohort will be stratified by CHD severity with enrichment for hypoplastic left heart syndrome and other single-ventricle defects. Primary outcomes include prevalent and incident hypertension, CKD, and kidney failure. The study will feature comprehensive phenotyping of kidney health through standardized clinical assessments, echocardiographic characterization, ambulatory blood pressure monitoring, and novel biomarker discovery using both targeted and untargeted proteomic approaches. A biorepository will be established to facilitate future ancillary studies.

**Significance:**

This study addresses critical knowledge gaps in pediatric cardio-nephrology by providing the first comprehensive and collaborative longitudinal assessment of kidney outcomes in children with CHD-a population largely excluded from existing pediatric nephrology cohorts. The study aims to inform risk stratification, enable early detection strategies, and guide development of targeted interventions to preserve long-term kidney health in this vulnerable and growing population.

## Introduction

### The growing population of children with CHD

Congenital heart disease (CHD) is the most common birth defect, affecting approximately 2.4 million individuals in the United States-including 1 million children and 1.4 million adults [1,2]. One in four children with CHD undergoes cardiac surgery, and with advances in surgical techniques and perioperative care, over 90% now survive to adulthood. This remarkable improvement in survival has shifted clinical focus toward understanding and preventing long-term complications that affect quality of life and longevity.

### Kidney disease in CHD

Among these long-term complications, kidney disease has emerged as a critical yet under-recognized threat. Multiple studies demonstrate that individuals with CHD face substantially elevated risks of hypertension, chronic kidney disease (CKD), and premature death [3–6]. In adults with CHD, hypertension prevalence reaches 47%, while the risks of hypertension and CKD are increased 1.4-fold and 3.4-fold respectively compared to the general population [7,8]. These sobering statistics underscore an urgent need to understand when and how these complications develop.

### Evidence for childhood origins of kidney disease

Our previous work suggests that the kidney disease burden observed in adults with CHD has its origins in childhood. In pediatric CHD populations, we identified a high burden of albuminuria (8%), hypertension (12%-17%), and CKD (21%-30%) following cardiac surgery [3,6]. Children with hypoplastic left heart syndrome (HLHS) face particularly severe risks, with 35% developing hypertension and 9% progressing to kidney failure during long-term follow-up [3–5].

Several perioperative factors predict these adverse outcomes including age less than 3 months at surgery, surgical complexity, need for perioperative dialysis, and cumulative number of cardiac surgeries. These findings strongly suggest that the excess burden of kidney disease in adults with CHD begins during childhood, although the natural history remains poorly characterized [9,10].

### The care gap: Under-recognition and under-treatment

Despite compelling evidence of risk, kidney complications in children with CHD remain under-diagnosed and under-treated. In the pediatric TRIBE-AKI cohort, while 17% had hypertension and 13% had CKD, only 4% had been evaluated by a pediatric nephrologist [6]. This care gap is particularly concerning given that early intervention with RAAS inhibitors and lifestyle modifications can effectively control blood pressure and albuminuria, potentially limiting kidney disease progression [11].

### Challenges in diagnosis and monitoring

***Blood Pressure Assessment:*** Accurate blood pressure assessment in pediatric patients presents a unique challenge. Ambulatory blood pressure monitoring (ABPM), the gold standard for pediatric hypertension diagnosis, captures circadian patterns and identifies masked hypertension, conditions that clinic measurements miss [12]. Our pilot study revealed ABPM abnormalities in 11 of 23 children with CHD, the most common being loss of nocturnal blood pressure dipping [13]. These findings highlight the inadequacy of routine clinic measurements for comprehensive cardiovascular risk assessment.

***High-Risk Populations:*** Children with single-ventricle physiology, particularly those with HLHS undergoing Fontan palliation, require special consideration. Worldwide there are now over 47,000 patients living with a Fontan circulation and aging into adulthood. Their unique hemodynamics characterized by elevated central venous pressure, reduced cardiac output, and neurohormonal activation place them at risk for kidney injury. Understanding how these physiological derangements affect kidney health is essential for developing targeted interventions to prevent premature morbidity and mortality.

### Beyond traditional risk factors

***Biomarker Innovation*:** Traditional kidney function assessment using serum creatinine and albuminuria provides limited insight into ongoing injury and repair processes. Novel biomarkers reflecting diverse pathways—glomerular health, tubular injury, inflammation, and fibrosis—offer opportunities for earlier detection and mechanistic understanding. Recent advances in proteomic technology now enable measurement of over 10,000 proteins potentially identifying mechanistic pathways and therapeutic targets.

***Genetic Contributions*:** While genetic factors contribute to approximately 40% of CHD cases, their role in kidney disease susceptibility remains unexplored [14–17]. In a systematic review, we identified 47 genes that could be pathogenic or highly probable risk factors potentially linking CHD to kidney disease, including 11 genes encoding ciliary proteins, structures critical for both cardiac and kidney development [17–21]. Genetic testing is often clinically available, and these data may provide important contextual data for understanding risk of CKD and identify subgroups for closer monitoring.

### The imperative for action

To date, no prospective studies have comprehensively characterized the incidence, progression, and mechanisms of kidney disease in children with CHD beyond 5 years post-surgery. This knowledge gap impedes development of screening protocols, risk stratification tools, and preventive interventions.

To address these critical gaps, as a team of pediatric nephrologists, cardiologists, and epidemiologists, we designed a multi-center prospective cohort study to provide the first comprehensive assessment of kidney and cardiovascular health trajectories in children with CHD. By integrating clinical phenotyping, advanced imaging, and novel biomarker discovery, we aim to transform the understanding and management of kidney disease in this vulnerable population.

## Methods

### Study design

**Fig 1:**
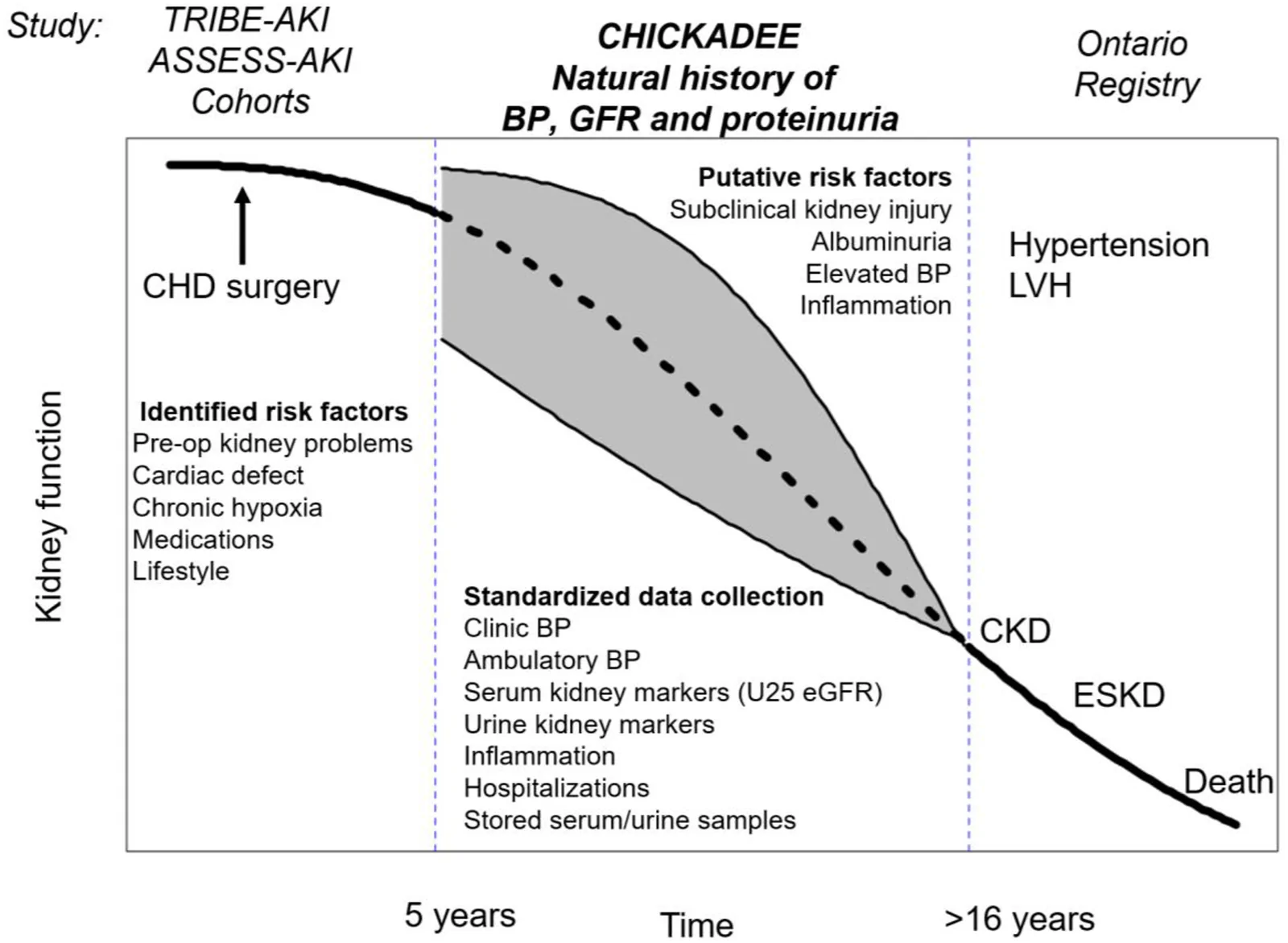
Schematic of known and hypothesized change in kidney function over time (not to scale). The left side of the figure describes findings from the TRIBE-AKI and ASSESS-AKI cohorts; the right side describes findings from the Ontario registry. The dashed line represents hypothesized change over time, with the gray area depicting uncertainty that will be investigated in the proposed CHICKADEE cohort.

**Fig 2:**
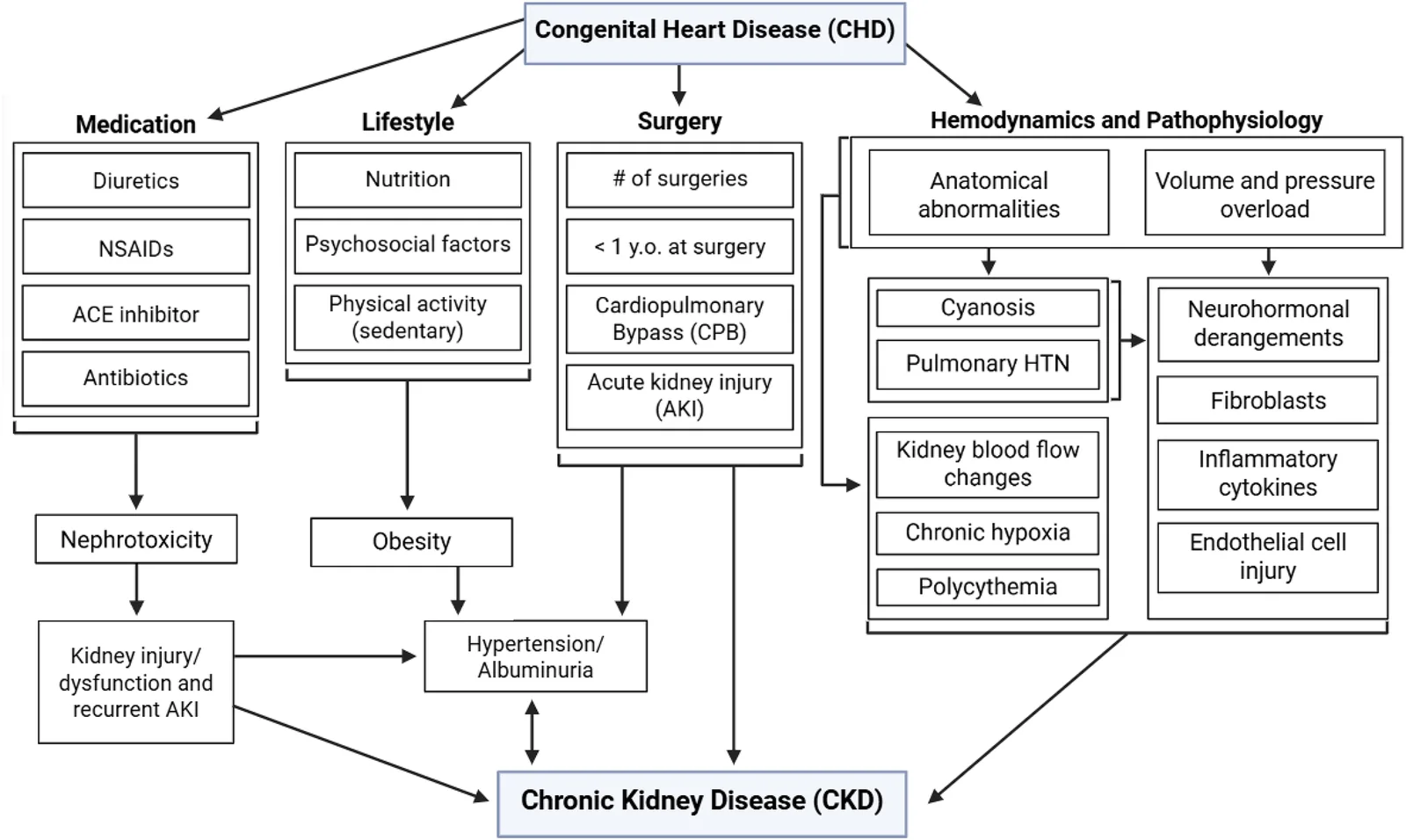
Conceptual framework of hypertension, albuminuria, and CKD in children with congenital heart disease: risk factors and mediators associated with the pathophysiology of kidney outcomes. ACE, angiotensin-converting enzyme; CKD, chronic kidney disease.

This observational cohort study aims to characterize the natural history of hypertension and kidney disease and identify traditional and novel baseline, peri-, and postoperative risk factors of hypertension and CKD (Fig 1). The CHICKADEE (**C**ongenital **H**eart disease **I**n **C**hildren: **K**idney-**A**ssociate**D** Conditions with **E**pidemiologic **E**ndpoints) cohort will offer the opportunity to define hypertension using 24-hour ambulatory blood pressure monitoring (ABPM) and more sensitive measures of glomerular and tubular health, injury, and function based on novel biomarkers. Other clinical information that may contribute to the development of hypertension and kidney disease will be collected to allow for risk factor identification unique to this patient population (Fig 2). We plan to incorporate measurements of neurohormonal and physiological markers to better understand the pathophysiology of kidney disease after cardiac surgery. Upon completion, CHICKADEE will help further elucidate the mediators and pathophysiology of kidney disease in CHD and provide a resource for important questions not yet conceived.

**Fig 3:**
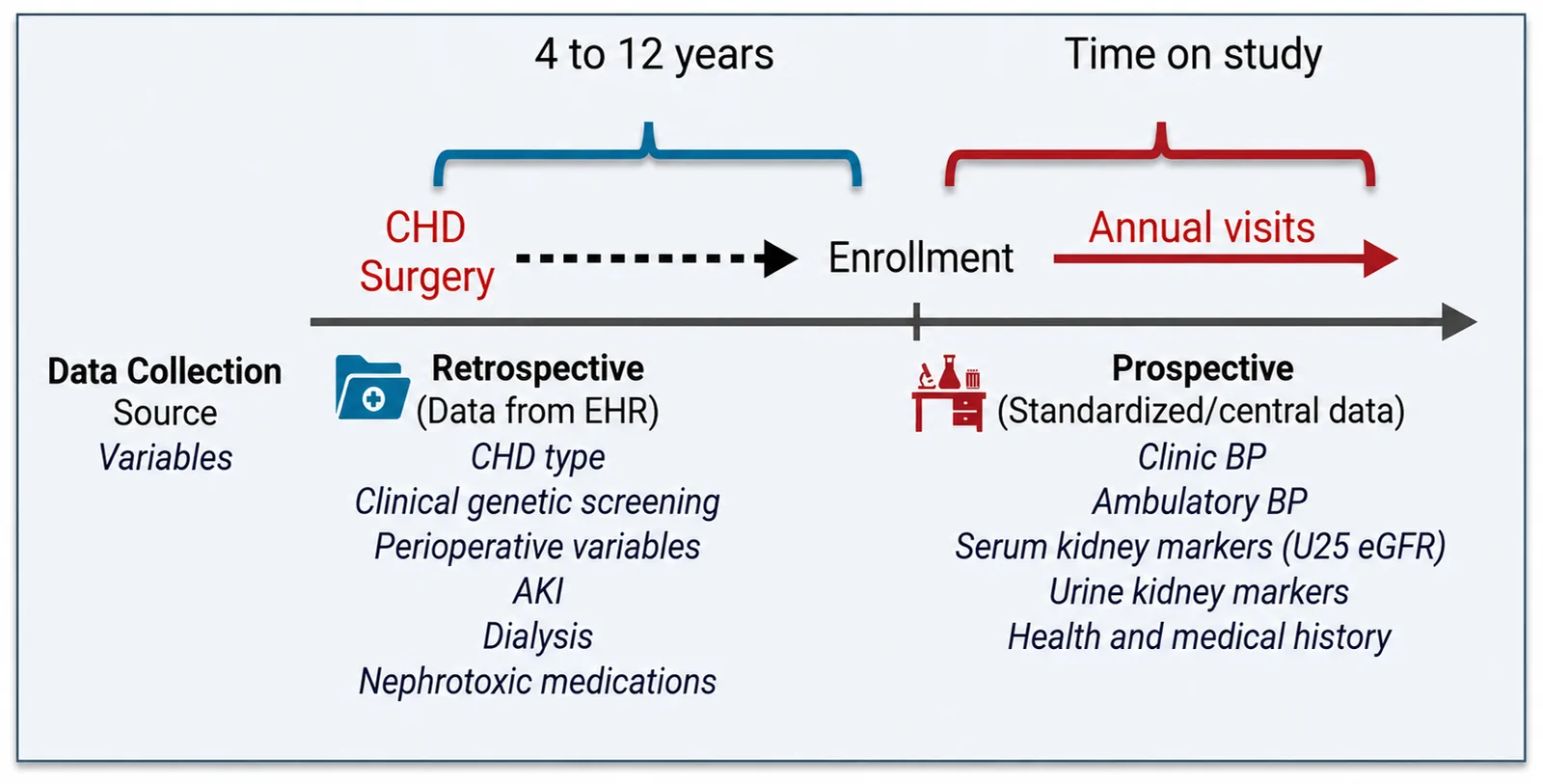
Schematic of CHICKADEE study design with a combination of retrospective data collection of perioperative variables and prospective data collection.

The CHICKADEE study is unique in that it offers both retrospective and prospective elements to gain novel insights into kidney health and injury in congenital heart disease, designed to provide longitudinal follow up for each enrolled patient (Fig 3). Briefly, CHICKADEE will have an interval cohort study design comprising an initial (baseline) visit and additional annual follow-up visits. Detailed retrospective data extracted from electronic medical records will complete the picture of CHD characteristics prior to enrollment.

### Study organization

The CHICKADEE study comprises a partnership of pediatric nephrologists, pediatric cardiologists, epidemiologists and research coordinators at three clinical sites, a coordinating center and central laboratory. The three clinical sites are located at Yale University (New Haven, CT), Cincinnati Children’s Hospital Medical Center (Cincinnati, OH) and Seattle Children’s Hospital (Seattle, WA). The Data Coordinating Center (DCC) and Central Lab are located at Johns Hopkins University (Baltimore, MD). The study website for CHICKADEE is located at chickadeestudy.com.

The clinical sites recruit and administer protocols to consenting patients who are identified as receiving regular pediatric cardiology care. Written consent is obtained from parents or guardians with assent obtained from children when appropriate. The DCC interfaces with the single IRB at the Johns Hopkins School of Medicine with reliance agreements for clinical sites. The DCC develops and maintains data collection systems through REDCap, manages and cleans data and conducts analyses for scientific studies. In addition, the DCC tracks recruitment efforts, receives, integrates and transmits information to the sites on study progress.

### Cohort participants

The CHICKADEE study has a targeted recruitment of 300 children, 4–16 years old, 4 to 12 years after their first cardiac surgery requiring bypass. Of the 300 participants, we aim for at least 15% (45 of 300) with single ventricle cardiac lesion and at least 35% (105 of 300) with a history of cyanotic CHD, approximately evenly distributed at each site. Table 1 details the complete inclusion and exclusion criteria for enrolling study participants. Recruitment will encompass children across a wide range of eGFRs at the time of enrollment, but patients with advanced CKD, specifically eGFR < 20 ml/min/1.73m^2^, or end stage kidney disease (ESKD) will not be included. We will not exclude children with congenital anomalies of the kidney and urinary tract (CAKUT), as CAKUT is more common in children with CHD. Additionally, exclusion criteria are history of non-bypass surgery, those who have received a heart transplant, those who are supported with a ventricular assist device, or those listed for transplant. These patients are ineligible due to the distinct physiology and medication profiles. We also excluded children who are 3 months or less after a recent cardiac surgery or children with no information available on their ‘index surgery,’ the first surgery on cardiopulmonary bypass. The start of the recruitment period for this study was March 1, 2025. We estimate that participant recruitment and data collection will be completed by December 2027. We estimate that results are expected by June 2028.

**Table 1:** Inclusion and exclusion criteria for the CHICKADEE study.

Inclusion criteria	Exclusion criteria
Pediatric participants aged 4 years to 16 years	Inability to provide informed or surrogate consent
First surgical repair of CHD 4 years to 12 years prior to cohort entry	Children with patent ductus arteriosus ligation, but no other cardiac surgery
Diagnoses of any CHD requiring surgical repair	Pre-existing advanced CKD (eGFR < 20 mL/min/1.73m^2^)
	Kidney failure (receipt of chronic dialysis or a kidney transplant)
	Comorbidities of glomerular kidney diseases, such as IgA nephropathy, Alport syndrome, or systemic lupus erythematosus nephritis

### Disease severity measures

The CHICKADEE study will use the Society of Thoracic Surgeons/European Association for Cardio-Thoracic Surgery (STAT) score to classify the complexity of cardiac surgery as 1, 2, 3, 4, or 5. The severity of CHD is defined using consensus guidelines. Severe CHD includes atrioventricular septal defects, tetralogy of Fallot, univentricular heart, transposition complex, truncus arteriosus, HLHS, and other single-ventricle cardiac lesions. We will also study the severity of CHD based on the presence or absence of aortic arch interruption.

Kidney function and injury will be measured using standardized renal panels measured at the central biochemistry and biomarker laboratory at Johns Hopkins. Specifically, GFR will be estimated using the recent “Under 25” (U25) equations validated for children and young adults at lower GFR levels and based on serum creatinine and cystatin C [21–25]. In addition, urinary markers of kidney injury and health, including albuminuria (i.e., urine albumin concentration), will be integrated with categories of GFR to identify CKD risk stages [26].

### Collection of study data

Following enrollment, we will collect a cardiovascular and kidney profile to determine the prevalence and severity of hypertension, albuminuria, and kidney health. Participants or parents complete a health questionnaire including documentation of medications they take. They have blood pressure measured using a standardized protocol, an ABPM device placed, and lab collection including urine and blood.

Retrospective medical record data will be collected on hypertension, albuminuria, and CKD (when available). Additionally, site coordinators will extract perioperative data for all surgical procedures from electronic medical records. Relevant data will include general clinical information and surgery-specific data such as duration of cardiopulmonary bypass, incident AKI, medications, and other adverse experiences during each hospital stay.

To characterize each participant’s clinical course from the time of their first surgery through enrollment in the study, detailed information will be collected including laboratory measurements, imaging, episodes of AKI, medications, all surgical interventions, and other treatments. Comprehensive information on life history, social background, access to care, and demographics will also be gathered as outlined in Table 2.

**Table 2:** Study components for CHICKADEE study.

Birth history (pre-surgery data)	• Birth weight • Prematurity status • Medication exposures • Serum creatinine data (from birth to time of index surgery)
Socio-environmental characteristics	• Environmental tobacco smoke exposure (screening questionnaire at annual visit) • Air pollution index (based on the latitude-longitude coordinates of participant’s residential address at the time of the baseline visit) • Type of health insurance • Highest attained parental education • Percent uninsured population in participant’s county of residence • Residential county type
Medications of interest	• Nonsteroidal anti-inflammatory drugs • Potassium-sparing diuretic agents • Loop diuretic agents • Thiazide diuretic agents • Angiotensin-converting enzyme inhibitors • Angiotensin 2 receptor blockers • Beta-blockers • Sacubitril/valsartan • Anticoagulants • Digoxin • Sotalol • Amiodarone • Flecainide
Echocardiographic features	1) Any left ventricular systolic dysfunction. 2) Moderate or severe left ventricular systolic dysfunction. 3) Moderate or severe systemic atrioventricular valve regurgitation. 4) Moderate or severe aortic insufficiency. We will use the definition of mild/moderate/severe dysfunction for all 4 features, as qualitatively assessed by the reading cardiologist in the report, regardless of the number.

Blood pressure measurement: Clinic BP is measured by auscultation using a standardized protocol with an appropriately sized cuff after the participant has been seated quietly for at least 5 minutes, with back supported and feet flat on the floor. Three measurements will be obtained using a validated manual device, and the average of the readings will be used for analysis.

Ambulatory BP monitor: ABPM is used following the recommendations in the 2022 American Heart Association Scientific Statement on ABPM in Children Ambulatory hypertension. Ambulatory hypertension will be defined as either mean systolic or diastolic BP > 95^th^ percentile (overall, wake, or asleep) based on the American Heart Association Scientific Statement on ABPM in Children [27]. Clinically measured BP with standardized instruments will provide clinic BP data to link with ABPM and establish specific BP phenotypes. The monitor (Spacelabs OnTrak) is placed on the child’s non-dominant arm and worn for 24 hours with BP readings taken every 20 minutes during the day and every 30 minutes overnight. Data will be downloaded and analyzed according to the guidance and BP thresholds in the 2022 AHA statement. ABPM studies will be considered adequate if they contain at least 41 BP measurements and one reading per hour over the monitoring period. We exclude children under 6 years of age or 120 cm in height from this analysis due to device inaccuracy.

Echocardiograms: Echocardiograms obtained close to each annual research visit are transferred to the central echocardiogram laboratory at Seattle Children’s Hospital for blinded interpretation by a pediatric cardiologist. Key features of interest will include left ventricular systolic dysfunction, left ventricular hypertrophy, left ventricular mass index, systemic atrioventricular valve regurgitation, ascending aortic and aortic root dilation, aortic insufficiency, aortic Doppler flow patterns, left atrial enlargement, and markers of diastolic dysfunction. Quantitative parameters will include ejection fraction (EF), with systolic dysfunction defined as EF < 55% and further categorized as mild (45–54%), moderate (30–44%), or severe (<30%). Cardiac structure will be assessed using z-scores normalized for body surface area.

### AKI Events

AKI will be defined by the KDIGO criteria: each participant’s hospitalization dates will be recorded to determine whether AKI should be classified as inpatient or outpatient and to differentiate hospitalized vs community-acquired AKI. Episodes of AKI will be classified as hemodynamic (kidney hypoperfusion based on the pattern of serum creatinine rise/fall, a negative fluid balance, and response to IV fluids), intrinsic AKI (based on the pattern of serum creatinine rise/fall, use of a contrast agent, response to IV fluids), or obstructive (based on kidney ultrasound findings and serum creatinine trends), or indeterminate.

### Biomarkers

The study aims to assess the viability of untargeted proteomic measurements and targeted biomarkers of injury and inflammation for characterizing subclinical kidney injury in the absence of changes in GFR. We will measure novel glomerular, tubular, and inflammatory biomarkers in the blood and urine sample obtained at study enrollment to assess the association with decline in kidney function and hypertension. We hypothesize that higher plasma TNFR1 and TNFR2, higher urine KIM-1, and lower urine EGF and uromodulin concentrations will be associated with primary outcomes. Assessing these biomarkers will capture the multifactorial pathophysiology of kidney health in children with CHD.

### Genetic risk factors

Genetic causes of CHD are clinically important and it is fundamental to characterize any syndromic or monogenic etiologies of CHD in this population, particularly to investigate putative additional risks related to CKD. Recent studies indicate that 60% of all CHD cases have no known genetic diagnosis, while 13% are syndromic and 15% are related to CNVs [28]. This makes genetic characterization of CHD cases in our study difficult. For complex CHD, patients with concern for dysmorphisms or a sequence of congenital anomalies, or when helpful in prenatal management, genetic testing is often conducted; this includes SNP array for chromosomal anomalies, and in recent years, sequence-based studies are used more often for improved detection of single gene disorders. Previous genetic testing data will be extracted from medical records and then stratified by syndromic diagnosis and detected CNVs to assess whether genetic variants are associated with increased risk of kidney-related complications.

### Outcomes

#### Hypertension.

The outcome of hypertension will be defined as the presence of abnormal ABPM or the use of antihypertensive agents to treat hypertension. ABPM staging will be quantified by the American Heart Association ABPM Guidelines based on mean 24 hour, daytime, and nocturnal systolic and diastolic blood pressure levels. High clinic blood pressure (i.e., elevated BP, Stage I or Stage II hypertension) will be defined using standardized protocols with stratification based on the 2017 AAP pediatric blood pressure guidelines [12]. The measurement of clinic blood pressure at the research study visit will be combined with the ABPM to determine one of 4 BP phenotypes: normotension (normal BP on both measures), white coat hypertension (normal ABPM and high clinic BP), masked hypertension (abnormal ABPM and normal clinic BP) and ambulatory hypertension (abnormal ABPM and high clinic BP). Circadian variation will be assessed using percent dipping of both systolic and diastolic BP.

#### CKD and eGFR decline.

CKD will be defined as eGFR < 90 mL/min/1.73 m^2^ or a urine albumin to creatinine ratio >30 mg/g. GFR will be estimated using standard pediatric equations based on age, sex, height, serum creatinine, and cystatin C [21,29]. The CHICKADEE study population will likely have higher GFR, on average, than the CKD populations typically used to develop pediatric eGFR equations. While this study will not use direct measures of GFR, measures of association (e.g., participants with a given surgical characteristic will have a 10% lower eGFR) are expected to be internally valid. Longitudinal eGFR measures will characterize the rate of eGFR change in mL/min/1.73 m^2^ per year or percent change per year from study enrollment to the last eGFR measured [30]. For purposes of characterizing changes, children who develop kidney failure will be imputed as having an eGFR of 10 mL/min/1.73m^2^. Methods to characterize non-normally distributed changes over time across groups of interest (e.g., diagnoses) will also be considered, as well as time to event analyses if there are enough events (e.g., kidney replacement therapy, incident hypertension) [30].

#### Microalbuminuria.

Microalbuminuria will be defined by the KDIGO guidelines as a urine albumin to creatinine ratio >30 mg/g.

### Power calculation

The study primarily seeks to quantify the incidence of comorbidities such as hypertension and proteinuria at least 4 years after first CHD surgery. We estimated the precision of p, which is the true rate of a condition of interest (e.g., hypertension, albuminuria, CKD), if the cohort size was 300 to ensure that we have at least p/2 precision. In other words, because the 95% confidence interval for p is $p\;\pm \;\text{1}.\text{9}\text{6}\sqrt{(p(\text{1}-p)/n}$, the precision for sample size n is determined by requiring $\text{1}.\text{9}\text{6}\sqrt{(p(\text{1}-p)/n}$ to be at least equal to or less than p/2 (e.g., if p is 0.05, n will yield a 95% CI from 0.025–0.075). For p = 20%, 10%, and 5%, the precision with 300 participants corresponds to 4.5%, 3.4%, and 2.5%. With n = 300, the 95% CI half-width equals p/2 at p ≈ 5% and is smaller than p/2 for p greater than 5%, which is the benchmark for the least acceptable precision. For p less than 5%, it becomes larger than p/2. According to previous findings in the TRIBE-AKI study enrolling only 131 children, 5 years after CHD surgery, 17% had hypertension, 8% had microalbuminuria and 1% had eGFR < 60 mL/min|1.73m^2^. We expect to have more outcomes because of the complementary time period after surgery, so a cohort of 300 children gives confidence that we can estimate the true rate of conditions with incidences as low as about 5% with strong precision.

## Discussion

There has been no systematic assessment of BP, albuminuria, and kidney function beyond 5 years after cardiac surgery in children. We previously documented that hypertension and CKD are common after cardiac surgery, but the risk factors and long-term trajectories of these outcomes are unknown. Recent findings in adults have shown that hypertension and CKD in childhood are independent risk factors for these same outcomes in adulthood and are associated with increased risk of mortality [31–33]. A detailed understanding of the incidence of hypertension, albuminuria, and CKD in CHD may allow strategies that target the early detection of hypertension and CKD. Earlier CKD detection after CHD surgery may prompt cardiovascular risk reduction interventions to promote cardiovascular health and improve quality of life.

Creation of the CHICKADEE cohort will demonstrate our ability to overcome traditional challenges in recruitment, sample collection, biomarker measurement, and outcome ascertainment in this understudied group of children at increased risk of kidney complications. This project will perform discovery research to better understand the development of hypertension and adverse kidney outcomes and use novel biomarkers to characterize the development of subclinical disease. This will allow for early identification, prompt referral to nephrology, and treatment of children at high risk of hypertension and CKD and provide new insights into the pathophysiology of kidney disease in children who have undergone surgery for CHD. The findings will facilitate improved clinical monitoring, stimulate hypotheses for clinical trials, and inform clinical management guidelines.
